# Indicator bacteria taxa reveal breeding site origins of *Aedes aegypti* mosquitoes

**DOI:** 10.1186/s13104-025-07482-y

**Published:** 2025-09-22

**Authors:** Katherine D. Mosquera, Olle Terenius

**Affiliations:** 1 Department of Cell and Molecular Biology, Biomedical Centre, Uppsala University, Uppsala, Sweden; 2 Present Address: Department of Molecular Biosciences, The Wenner-Gren Institute, Stockholm University, Stockholm, Sweden

**Keywords:** *Aedes aegypti*, Microbiota, Breeding sites, Indicator species

## Abstract

**Background:**

The microbiota of adult mosquitoes reflects both their life history and the environments they encounter. Linking specific bacterial communities in adult mosquitoes to their larval aquatic habitats would suggest that microbiota profiling can serve as a tool to identify the breeding site origins of mosquitoes.

**Objective:**

We investigated whether specific OTUs within the microbiota of female *Aedes aegypti* could serve as reliable indicators of their larval habitats, thereby identifying the breeding environments from which adult mosquitoes emerged.

**Conclusion:**

Indicator species analysis on a previously published dataset of *Ae. aegypti* mosquitoes collected from six different types of breeding sites in Puerto Rico revealed significant differences in microbial communities among mosquitoes emerging from distinct aquatic habitats. Specific OTUs showed high specificity and fidelity to their respective breeding sites. This included members of the families Erysipelotrichaceae and Cytophagaceae in mosquitoes from plastic buckets, Burkholderiaceae and Clostridiaceae in mosquitoes from trash cans, Actinomycetaceae in mosquitoes from discarded tires, Microbacteriaceae in mosquitoes from septic tanks, and Enterobacteriaceae in mosquitoes from tree holes. These findings suggest that the microbiota of adult mosquitoes retains markers of their larval habitats, which could be used to trace their origins and inform targeted mosquito control efforts.

**Supplementary Information:**

The online version contains supplementary material available at 10.1186/s13104-025-07482-y.

## Introduction

The microbiota found in adult vector mosquitoes partially reflects their life history. While the gut microbiota is influenced by physiological properties specific to each mosquito species [1], the overall microbial profile of adult mosquitoes is largely shaped by interactions with their environment. For example, previous research demonstrated that the microbial profile of individual adult mosquitoes captured in adjacent villages in Burkina Faso could accurately predict their village of origin with 90% probability [2]. Among the bacterial taxa most strongly associated with village differentiation based on Random Forest analysis, *Massilia* and *Shewanella* were assumed to originate from the larval habitats, as these genera are often found in freshwater environments. However, because the adult mosquitoes were captured indoors, their specific breeding sites remained unidentified.

Mosquitoes have been shown to drink water immediately after eclosion, thereby inoculating their gut with bacteria from their breeding site [3]. In a recent study by Caragata et al. [4], specialized emergence traps were used to collect adult *Aedes aegypti* mosquitoes within 24 h of eclosion. These traps were designed to ensure that mosquitoes could only drink from and come into direct contact with the water in which they had developed, isolating any container-specific bacteria. Importantly, the mosquitoes remained confined within the traps, preventing exposure to microorganisms from other sources. Mosquitoes were sampled from several types of breeding sites, including large plastic buckets, septic tanks, discarded tires, inground trash cans, tree holes, and water meter pits. In their study, beta-diversity measures represented in ordination plots of 16S amplicon sequences, grouped at the family level, showed a clear separation for two of the six breeding site types, while the remaining sites exhibited overlap and lacked clear distinction. However, their permutational multivariate analysis of variance (PERMANOVA) indicated significant differences in the microbial profiles of mosquitoes collected from the six different container types.

With these indications of the existence of specific microbial profiles, we decided to take the analysis a step further and explore whether individual operational taxonomic units (OTUs) could indicate which type of breeding site an individual female *Ae. aegypti* emerged from, in an indicator species analysis.

### Methodology

Considering that the PERMANOVA test in Caragata et al. [4] evidenced significant differences in the bacterial communities associated with *Ae. aegypti* mosquitoes emerging from different rearing containers (PERMANOVA: F = 2.2278, df = 5, R^2^ = 0.11467, *P* = 0.001), we deemed it relevant to search for indicator OTUs. For this, we used the publicly available community data matrix and metadata from Caragata et al. [4]. A total of 92 mosquito samples, emerging from six different types of breeding sites, were analyzed (Table 1). An indicator species analysis (ISA) was conducted to identify bacterial taxa that reflect the biotic and/or abiotic characteristics encompassed within each breeding container type. This analysis was performed in the R environment using the *indicspecies* package v1.7.7 [5].

**Table 1:** Number of adult *Aedes aegypti* mosquitoes collected from each type of breeding container

Container type (*N*)	Number of samples
Plastic buckets (4)	18
Septic tanks (3)	17
Tires (3)	15
Trash cans (3)	11
Tree holes (3)	16
Water meter pits (3)	15

**(N)** *denotes the number of containers sampled for each container type.*

## Results

The indicator species analysis revealed distinct bacterial taxa associated with each type of mosquito breeding site. Specifically, 139, 20, 31, 64, 4, and 20 OTUs were linked to plastic buckets, septic tanks, tires, trash cans, tree holes, and water meters, respectively. Table 2 presents the indicator taxa with high specificity and fidelity to the ecological conditions of each container type, containing OTUs with an indicator value index (IndVal) above 0.8. For a complete list of all indicator species, regardless of their IndVal, see Supplementary Table 1.

**Table 2:** OTUs identified in *Aedes aegypti* mosquitoes collected in different types of breeding containers in Puerto Rico

Container type	OTU ID	Specificity	Fidelity	Indicator Value	*P* value	Family	Genus
Plastic buckets	OTU 56	0.9901	0.9444	0.967	0.001	Erysipelotrichaceae	*Erysipelothrix*
	OTU 35	0.9276	1	0.963	0.001	Cytophagaceae	*Emticicia*
	OTU 14	0.925	1	0.962	0.003	Enterobacteriaceae	*Yersinia*
	OTU 65	0.9072	1	0.952	0.001	Pseudomonadaceae	*Pseudomonas*
	OTU 133	0.8021	1	0.896	0.001	Erysipelotrichaceae	*Bulleidia*
	OTU 103	0.9855	0.7778	0.876	0.001	Flavobacteriaceae	*Flavobacterium*
	OTU 168	0.9819	0.7778	0.874	0.001	Chitinophagaceae	*Sediminibacterium*
	OTU 6484	0.7574	1	0.87	0.001	Erysipelotrichaceae	*Erysipelothrix*
	OTU 111	0.961	0.7778	0.865	0.001	Bacillaceae	*Bacillus*
	OTU 50	0.737	1	0.858	0.001	Crenotrichaceae	*Crenothrix*
	OTU 40	0.7197	1	0.848	0.001	Xanthobacteraceae	*Ancylobacter*
	OTU 97	0.99	0.7222	0.846	0.001	Pseudomonadaceae	*Pseudomonas*
	OTU 63	0.714	1	0.845	0.001	Methylophilaceae	*Methylobacillus*
	OTU 272	0.8843	0.7778	0.829	0.001	Porphyromonadaceae	*Dysgonomonas*
	OTU 141	0.749	0.8889	0.816	0.001	Rhodobacteraceae	*Rhodobacter*
	OTU 340	0.9915	0.6667	0.813	0.001	Acetobacteraceae	*Roseomona*
	OTU 34	0.6574	1	0.811	0.001	Rhodocyclaceae	*Rhodocyclus*
	OTU 147	0.7358	0.8889	0.809	0.001	Erysipelotrichaceae	*Erysipelothrix*
	OTU 85	0.8407	0.7778	0.809	0.001	Burkholderiaceae	*Pandoraea*
	OTU 76	0.6856	0.9444	0.805	0.001	Synergistaceae	*Synergistes*
	OTU 74	0.6809	0.9444	0.802	0.001	Rikenellaceae	*Rikenella*
	OTU 72	0.6803	0.9444	0.802	0.001	Burkholderiaceae	*Polynucleobacter*
	OTU 203	0.7721	0.8333	0.802	0.001	Cytophagaceae	*Cytophaga*
Trash cans	OTU 7	0.9743	0.9091	0.941	0.004	Burkholderiaceae	*Leptothrix*
	OTU 78	0.9917	0.7273	0.849	0.001	Acidimicrobiaceae	*Aciditerrimonas*
	OTU 401	0.9782	0.7273	0.843	0.001	Thermoanaerobacteraceae	*Moorella*
	OTU 374	0.9759	0.7273	0.842	0.001	Conexibacteraceae	*Conexibacter*
	OTU 132	0.9607	0.7273	0.836	0.001	Clostridiaceae	*Clostridium*
Discarded tires	OTU 59	0.9292	0.9333	0.931	0.001	Actinomycetaceae	*Actinomyces*
	OTU 81	0.8841	0.8	0.841	0.002	Sutterellaceae	*Parasutterella*
	OTU 71	0.7839	0.8667	0.824	0.028	Pseudomonadaceae	*Pseudomonas*
	OTU 196	0.9257	0.7333	0.824	0.002	Xanthomonadaceae	*Stenotrophomonas*
Septic tanks	OTU 60	0.9233	0.7059	0.807	0.004	Microbacteriaceae	*Leucobacter*
Tree holes	OTU 474	0.8549	0.9375	0.895	0.04	Enterobacteriaceae	*Shigella*

Notably, plastic buckets contain more OTUs with an indicator value above 0.8 than all the other container types taken together (23 vs. 11). The top indicator taxa with IndVal > 0.8 belonged to the families Erysipelotrichaceae (plastic buckets), Burkholderiaceae (septic tanks), Actinomycetaceae (tires), and Microbacteriaceae (trash cans).

Table 2. OTUs identified in *Aedes aegypti* mosquitoes collected in different types of breeding containers in Puerto Rico.

## Discussion

The microbial communities associated with mosquitoes are largely influenced by the specific breeding sites where they develop. Understanding these associations is crucial for identifying environmental factors that contribute to mosquito vector competence and the spread of arboviral diseases. This study focuses on identifying and analyzing individual OTUs as indicators of the specific breeding sites from which female *Ae. aegypti* mosquitoes emerge. Since the collected mosquitoes had no exposure to external microbial sources, the bacteria identified are likely representative of their breeding environments, offering valuable insights into the ecological factors that shape mosquito-associated microbiota.

### Which are the top bacteria?

In this study, newly emerged adult mosquitoes were captured from various container types, with indicator species showing strong associations (IndVal above 0.8) identified in plastic buckets, trash cans, discarded tires, septic tanks, and tree holes.

Mosquitoes emerging from **plastic buckets** were uniquely associated with several members of the families Erysipelotrichaceae (OTU 56) and Cytophagaceae (OTU 35). These containers are known to be highly productive for the immature development of *Aedes* mosquitoes in urban centers of dengue-endemic areas [5]. In a study assessing the midgut microbiota of Colombian *Ae. aegypti* populations with varying levels of insecticide resistance, it was found that the order Erysipelotrichales was significantly enriched in resistant populations [6]. This is particularly noteworthy given that bacteria belonging to the genus *Erysipelothrix* are known as the etiological agents of the cutaneous diseases erysipelas in animals and erysipeloid in humans. The presence of Erysipelotrichaceae in *Ae. aegypti* mosquitoes from plastic buckets raises important considerations, especially since *Ae. aegypti* is capable of transmitting bacteria mechanically [7]. While opportunistic and potentially pathogenic bacteria associated with mosquito microbiota do not necessarily cause infections, mechanical vectors can spread various bacteria to different hosts. The risk of infection ultimately depends on the susceptibility of the host and the extent of contact with the bacteria [8]. Notably, the family Cytophagaceae has been identified as a core component of the saliva microbiota in *Aedes albopictus* [9] and has been recognized as a significant microbial biomarker for predicting infection of Dengue virus (DENV) in this mosquito species [10]. Additionally, bacteria from the genus *Emticicia* (in 2024 moved from the family Cytophagaceae to the new family Leadbetterellaceae; [11]) have been found in water samples and *Ae. aegypti* larvae collected in French Guiana [12]. The detection of *Emticicia* in both larvae and recently emerged adults suggests that these bacteria are consistently associated with *Ae. aegypti* from the larval stage to adulthood. This could indicate that *Emticicia* either persists through the mosquito metamorphosis or is acquired anew during or after emergence.

Additionally, *Pseudomonas* (Pseudomonadaceae) was identified as an indicator genus in mosquitoes reared in plastic buckets (OTUs 65 and 97) and discarded tires (OTU 71). This genus is frequently found in the microbiota of *Aedes* mosquitoes, colonizing not only the midgut but also salivary glands and reproductive tissues, indicating a broad capacity for tissue colonization [13]. The association of *Pseudomonas* with artificial breeding containers may reflect an ecological advantage in nutrient-rich, low-oxygen environments typical of urban habitats. Notably, *Pseudomonas sp.* has been detected in *Ae. aegypti* populations resistant to temephos and deltamethrin, suggesting a possible role in insecticide resistance under field conditions [14]. Complementarily, experimental studies in *Anopheles gambiae* have demonstrated that larval exposure to *Pseudomonas* strains results in systemic infections that persist into adulthood and significantly increase survival after permethrin exposure, likely through insecticide degradation or modulation of host detoxification pathways [15]. These findings highlight the ecological and physiological relevance of *Pseudomonas* in vector populations, particularly in urban environments where both pesticide pressure and human-made breeding containers are common.

Mosquitoes that emerged from **trash cans** were found to be associated with the bacterial genus *Leptothrix* (OTU 7). This genus has been previously identified as a common microbial inhabitant in both male and female *Ae. aegypti* mosquitoes [16]. Notably, *Leptothrix* has also been reported in association with *Lutzomyia longipalpis* [17], another important insect vector, suggesting a broader ecological relevance of this bacterial genus across different vector species. In addition, *Leptothrix* bacteria have been implicated as gut microorganisms responsible for the degradation of plastic particles in the beetle *Tenebrio molitor* [18]. The association of *Leptothrix* with *Ae. aegypti* mosquitoes, particularly those emerging from environments rich in plastic waste such as trash cans, suggests a potential ecological interaction with important implications. These bacteria may aid mosquitoes in digesting or breaking down complex materials like plastic, which are prevalent in their breeding habitats. This relationship could confer an adaptive advantage to mosquitoes in urban environments, where plastic pollution is widespread. Furthermore, another indicator taxon associated with mosquitoes emerging from trash cans is the genus *Clostridium* (OTU 132). These bacteria are particularly notable as the only obligate anaerobic microorganisms identified to date in both the larval and adult stages of *Ae. aegypti* [19, 20].

Members of the family Actinomycetaceae (OTU 59) were identified in mosquitoes developing in **discarded tires**, a habitat often characterized by harsh and polluted conditions. Previous studies have isolated bacteria from this taxon in field-collected mosquitoes, such as *Culex gelidus* [21] and *Ae. albopictus* [22]. Interestingly, in a study on black soldier fly larvae, an enrichment of Actinomycetaceae was observed when the larvae were exposed to plastic substrates. This enrichment was associated with functional shifts within the gut microbiome, which facilitated the degradation of complex polymers, potentially mitigating the negative effects of these pollutants on the larvae [23]. A similar process could be hypothesized in mosquitoes developing in discarded tires. Actinomycetaceae bacteria might be pivotal in aiding mosquitoes to thrive in environments laden with leachates from rubber and other pollutants commonly found in discarded tires. By promoting the degradation of complex polymers, Actinomycetaceae bacteria could help sustain gut health and enhance digestive efficiency, thereby supporting nutrient absorption in conditions that are otherwise nutrient-poor or chemically challenging.

Bacteria from the genus *Stenotrophomonas* (OTU 196) were also identified as an indicator genus in mosquitoes collected from discarded tires. This genus is widely recognized as part of the mosquito microbiota and has been detected in several tissues, including the midgut, reproductive organs, and salivary glands of *Aedes* mosquitoes [13, 20]. Its ability to colonize multiple internal tissues suggests a stable association that may extend beyond transient environmental acquisition.

In mosquitoes emerging from **septic tanks**, the presence of the indicator taxon *Leucobacter*, a member of the Microbacteriaceae family (OTU 60), can be attributed to the strong association of these bacteria with larval habitats rich in organic matter. Microbacteriaceae are often abundant in mosquito larvae, particularly in environments that are nutrient-dense and potentially polluted, such as septic tanks [24]. These conditions likely support the growth and persistence of *Leucobacter* during the larval stage. However, studies have shown that the abundance of Microbacteriaceae is significantly reduced during the metamorphosis [25], leading to their lower abundance in adult mosquitoes [19, 26]. The detection of *Leucobacter* in newly emerged adults may therefore reflect a temporary phase during which microbial leftovers of the immature stages persist before the microbiota of the adult fully stabilizes as the insect adapts to new diets and environmental conditions. This indicates that *Leucobacter* could play a role during the final stages of mosquito development, potentially influenced by the specific conditions of the breeding site, before its abundance diminishes as the mosquito ages [26].

For mosquitoes emerging from **tree holes**, the genus *Shigella* (OTU 474) was identified as a representative taxon. Although specific information on *Shigella* interactions with *Aedes* mosquitoes is limited, insights can be gained from studies on the broader *Escherichia*-*Shigella* group. *Escherichia-Shigella* has consistently been identified in the microbiota of *Ae. aegypti* and *Ae. albopictus*, particularly in the salivary glands and gut [20, 27, 28]. This suggests that these bacteria may be a stable component of the mosquito microbiota, potentially playing a role in the physiological processes of the host and influencing its ability to transmit pathogens. The presence of *Escherichia-Shigella* in the mosquito gut is particularly noteworthy, as it may contribute to nutrient competition or modulate immune responses in ways that affect the replication and transmission of arboviruses. Indeed, *Shigella* sp. has been identified as part of the mosquito microbiota, contributing to host immunity and metabolism, which influences DENV susceptibility in *Ae. albopictus* [10]. Although specific research on *Shigella* is limited, general observations indicate that *Escherichia-Shigella* bacteria can significantly influence mosquito vector competence. For example, Dickson et al. [29] demonstrated that larval exposure to an Enterobacteriaceae isolate, potentially including *Shigella*, led to decreased antibacterial activity in the hemolymph of adult *Aedes aegypti* mosquitoes and reduced DENV dissemination titers. These findings establish a functional link between larval exposure to environmental microbes and carryover effects on adult mosquito traits that are critical for vectorial capacity. Bacteria like *Shigella* may thus shape the immune system of the mosquito during development, potentially altering its ability to transmit arboviruses as an adult.

### How can the information be used?

We found strong evidence supporting the conclusion that adults emerging from a specific type of breeding site have a distinct microbial profile. This suggests that adults, regardless of where they are captured, but sharing a similar microbiota, likely originate from the same type of larval habitat. The ability to connect an adult mosquito to its breeding site opens up for targeted control, which could be implemented in surveillance strategies that focus on breeding sites and locations that represent a high risk to human populations. In addition, there are bacterial taxa that have been reported as suitable symbiotic partners positively impacting mosquito history traits and pathogen transmission. Identifying bacteria associated with *Ae. aegypti* emerging from specific types of breeding sites would aid to better understand the productivity of mosquito aquatic niches, as well as hot spots for the spread of epidemic arboviral diseases.

### Limitations

Microbial communities can vary significantly over time and across different geographical points. Given this, the findings presented in this study might be specific to the time and location where the samples were collected, limiting the applicability of our results to other contexts. Longitudinal studies of the microbial communities in water and mosquitoes could provide new insights into the temporal dynamics of the microbiota in specific locations. Such studies may reveal patterns of stability or change, enhancing our understanding of the factors influencing microbial community structure over time. Thus, while we here can show that adult mosquitoes within 24 h of emergence have members of their microbiota that uniquely indicate their breeding sites, we have no data on how persistent these microbial signatures are in the adult mosquitoes.

Identifying indicator OTUs provides information on the presence of specific bacterial taxa, but does not necessarily reveal their functional roles or ecological significance. Further studies would be needed to understand how these microbes interact with the mosquito host and the environment.

## Supplementary Information

Below is the link to the electronic supplementary material.

Supplementary Material 1

## Data Availability

All data supporting the findings of this study are available in Supplementary Table 1.

## Abbreviations

PERMANOVA: Permutational multivariate analysis of variance
OTU: Operational taxonomic unit
ISA: Indicator species analysis
IndVal: Indicator value index
DENV: Dengue virus
